# Expanding the RpoS/σ^S^-Network by RNA Sequencing and Identification of σ^S^-Controlled Small RNAs in *Salmonella*

**DOI:** 10.1371/journal.pone.0096918

**Published:** 2014-05-08

**Authors:** Corinne Lévi-Meyrueis, Véronique Monteil, Odile Sismeiro, Marie-Agnès Dillies, Marc Monot, Bernd Jagla, Jean-Yves Coppée, Bruno Dupuy, Françoise Norel

**Affiliations:** 1 Institut Pasteur, Unité de Génétique Moléculaire, Département de Microbiologie, Paris, France; 2 CNRS, ERL3526, Paris, France; 3 Université Paris Sud XI, Orsay, France; 4 Institut Pasteur, Plate-forme Transcriptome et Epigénome, Département Génomes et génétique, Paris, France; 5 Institut Pasteur, Laboratoire Pathogenèse des bactéries anaérobies, Département de Microbiologie, Paris, France; University of Padova, Medical School, Italy

## Abstract

The RpoS/σ^S^ sigma subunit of RNA polymerase (RNAP) controls a global adaptive response that allows many Gram-negative bacteria to survive starvation and various stresses. σ^S^ also contributes to biofilm formation and virulence of the food-borne pathogen *Salmonella enterica* serovar Typhimurium (*S*. Typhimurium). In this study, we used directional RNA-sequencing and complementary assays to explore the σ^S^-dependent transcriptome of *S*. Typhimurium during late stationary phase in rich medium. This study confirms the large regulatory scope of σ^S^ and provides insights into the physiological functions of σ^S^ in *Salmonella*. Extensive regulation by σ^S^ of genes involved in metabolism and membrane composition, and down-regulation of the respiratory chain functions, were important features of the σ^S^ effects on gene transcription that might confer fitness advantages to bacterial cells and/or populations under starving conditions. As an example, we show that arginine catabolism confers a competitive fitness advantage in stationary phase. This study also provides a firm basis for future studies to address molecular mechanisms of indirect regulation of gene expression by σ^S^. Importantly, the σ^S^-controlled downstream network includes small RNAs that might endow σ^S^ with post-transcriptional regulatory functions. Of these, four (RyhB-1/RyhB-2, SdsR, SraL) were known to be controlled by σ^S^ and deletion of the *sdsR* locus had a competitive fitness cost in stationary phase. The σ^S^-dependent control of seven additional sRNAs was confirmed in Northern experiments. These findings will inspire future studies to investigate molecular mechanisms and the physiological impact of post-transcriptional regulation by σ^S^.

## Introduction

In eubacteria, a single multi-subunit RNA polymerase (RNAP) is responsible for transcription. Although the core RNAP (E, α2ββ’ω) is capable of transcript elongation and termination, it cannot specifically initiate transcription from a promoter site. Promoter recognition relies on an additional subunit, σ, which associates with E to form the holoenzyme Eσ [1]. σ directs RNAP to specific promoters, is involved in promoter melting, and dissociates stochastically once sequence-specific promoter DNA contacts are no longer required. All bacteria have a primary house-keeping sigma factor, known as σ^70^ (RpoD) in *Escherichia coli* (*E. coli*) and *Salmonella*, which promotes the transcription of genes required for the essential functions in the cell. Most bacteria also have one or more alternative σ factors that direct transcription of specific subsets of genes [1]. The alternative sigma factor σ^S/38^ (RpoS) controls a global adaptive response allowing many Gram-negative bacteria to survive nutrient deprivation and environmental stresses [1]–[3]. σ^S^ also contributes to virulence and biofilm formation of *Salmonella enterica* serovar Typhimurium (*S*. Typhimurium) [3]–[5], a wide host-range pathogen and a major cause of human gastroenteritis and foodborne disease.

Previous works have focused on the complex regulation of *rpoS* in *E. coli* K-12 and on σ^S^ promoter specificity [2], [3]. In contrast to σ^70^, σ^S^ is almost undetectable in early exponential phase and is induced in stationary phase or in response to various stresses by a fine-tuned combination of transcriptional, translational and proteolytic controls [2], [3]. σ^S^ and σ^70^ bind to almost identical –35 and –10 promoter elements, a finding consistent with the high degree of sequence similarity between these two sigmas in their DNA binding regions [3], [6]. The activity of Eσ^S^ and Eσ^70^ holoenzymes can be modulated by additional regulatory proteins that bind to the promoter regions and can also contribute to σ factor selectivity at a given promoter [2], [3].

σ^S^ regulons have been characterized using microarrays in *E. coli* and occasionally in other bacterial species [3], [7], [8], [9], but not in *Salmonella*. Indeed, previous transcriptional profiling using a *S*. Typhimurium *rpoS* mutant only focussed on σ^S^-activated genes requiring σ^E^ for maximal expression [10]. More than 10% of the *E. coli* genes were found to be under positive control by σ^S^ [3]. In addition, negative effects of σ^S^ on gene expression is an important but poorly understood aspect of σ^S^-dependent control in *E. coli* [2], [3], [7]. Elimination of these negative effects in *rpoS* mutants likely contributes to the growth advantage of these mutants in some environments in the absence of stress [11], [12]. Our previous studies suggest that σ^S^ exerts negative effects on gene expression and growth capabilities in *Salmonella* as well [13]–[15], although it is not known to which extent.

In this study, we used directional RNA-sequencing and complementary assays to explore the σ^S^-dependent transcriptome of *S*. Typhimurium. Our data confirm the large impact of σ^S^ on gene transcription in stationary phase bacteria, including gene repression by σ^S^, and provide insights into the main physiological functions of σ^S^ in *S*. Typhimurium. Further, we show that the σ^S^-controlled downstream network includes small RNAs that might endow σ^S^ with post-transcriptional regulatory functions and might be intermediate regulators in the down-regulation of gene expression by σ^S^. This study provides a firm basis for future studies to address molecular mechanisms used by σ^S^ to control gene expression indirectly and to assess the physiological impact of negative regulation by σ^S^.

## Materials and Methods

### Bacterial Strains, Bacteriophage and Growth Conditions

Strains are listed in Table S1. Bacteriophage P22HT105/1*int* was used to transfer mutations and *lacZ* fusions between *Salmonella* strains by transduction [16]. Green plates, for screening for P22-infected cells or lysogens, were prepared as described previously [17]. Bacteria were routinely grown in Luria-Bertani medium (LB) [18] at 37°C under aeration. Antibiotics were used at the following concentrations (in µg per ml): carbenicillin (Cb), 100; kanamycin, (Km) 50; and tetracycline (Tet) 20.

### DNA Manipulations and Inactivation of Chromosomal Genes

Standard molecular biology techniques were used [4], [18]. Oligonucleotides were obtained from Sigma-Aldrich and are listed in Table S2. DNA sequencing was performed by Beckman Coulter Genomics. Chromosomal deletions in the *sdsR, sraL* and *csrC* loci of *Salmonella* ATCC14028 were generated using *tetAR* PCR-generated linear DNA fragments (Table S2) and the λ-Red recombination method [19], [20]. The scarless in frame deletion of *rpoS* in strain VFC331 was achieved with a two-step Red-recombinase-based recombineering procedure [20]. The procedure involves 1) replacement of the *rpoS* coding sequence by a *tetAR* module (produced by PCR, Table S2) and 2) replacement of the *tetRA* module by a DNA fragment obtained by PCR (Table S2) and carrying the desired deletion through positive selection of tetracycline-sensitive recombinants [21]. All strains were confirmed to contain the expected mutation by DNA sequencing. Transcriptional *lacZ* fusions in the *astA, katE* and *katN* genes were previously described [14], [22].

### Isolation of Total RNA from *S*. Typhimurium

Total RNA was isolated from cells grown aerobically until late stationary phase (18 h growth) in LB at 37°C, using TRIzol. Pellets of cells were resuspended in 12.5 mM Tris-HCl (pH 7.6), 10 mM EDTA, 10% glucose. After addition of 1/5 volume of 0.5 M EDTA, disruption of cells was performed by vigorous shaking using glass beads (G1277, Sigma-Aldrich) in acid phenol pH 4.5 (Interchim). After centrifugation, the aqueous phase was carefully mixed with 2 volumes of TRIzol (Invitrogen), and five minutes later with a chloroform-isoamyl alcohol mixture (24∶1). After centrifugation, chloroform-isoamyl alcohol was added to the aqueous phase and the mixture was allowed to stand for five minutes before centrifugation. Total RNA present in the aqueous phase was precipitated with isopropanol. After centrifugation, the pellet was washed in 70% Ethanol, air-dried and resuspended in RNAse-free water. The RNA was subsequently treated with DNaseI (Ambion) and its quality was analyzed using an Agilent BioAnalyzer.

### cDNA Library Preparation, Sequencing and Analysis of Sequences

Total RNA from three biological replicates of each strain was isolated from late stationary phase cultures as described above and its quality checked with an Agilent BioAnalyzer. Starting from 10 µg of total RNA, rRNA content was depleted using MicrobExpress kit (Ambion). The rRNA depleted fraction was used for construction of strand specific single end cDNA libraries according to manufacturer’s instructions (using Truseq Small RNA sample prep kit, Illumina). Libraries were sequenced using an Illumina Hiseq2000 sequencer (multiplexing 3 samples per lane) according to manufacturer’s instructions (Illumina). Sequences were demultiplexed using the Illumina pipeline (Gerald, included in CASAVA version 1.7) giving FASTQ formatted reads. Those reads were cleaned from adapter sequences and sequences of low quality using an in-house program. Only sequences with a minimum length of 30 nucleotides were considered for further analysis. Bowtie [23] (version 0.12.7, –chunkmbs 200, -m 50, -e50, -a –best, –solexa1.3-quals) was used to align to the reference genome (CP001363.1 and CP001362.1). HTseq-count (Simon Anders, www-huber.embl.de/users/anders/HTSeq/doc/count.html, parameters: -m intersection-nonempty, -s yes, -t gene) was used for counting genes. Statistical analyses were performed using R version 2.15.1 [24] and Bioconductor packages (http://www.bioconductor.org/). Genes with nul raw counts in all samples were excluded from the data table. Normalization and differential analysis were performed using DESeq version 1.8.3 [25]. The whole dataset was first normalized using the normalization function of DESeq and dispersion was estimated with default parameters. The statistical test was then applied on pairs of strains. Resulting p-values were adjusted for multiple comparisons according to the BH method [26]. Two significance thresholds (0.05 and 0.001) were applied on adjusted p-values in order to declare genes as differentially expressed. The mapped reads were formatted into graph files for visualization using COV2HTML [27] (https://mmonot.eu/COV2HTML) and GBrowse (http://genopole.pasteur.fr/gbrowse/).

### RNA-seq Transcriptome Accession Number

The RNA-seq data discussed in this publication have been deposited in NCBI’s Gene Expression Omnibus and are accessible through GEO Series accession number GSE46380 (http://www.ncbi.nlm.nih.gov/geo/query/acc.cgi?acc=GSE46380).

### Analysis of sRNAs Expression from RNAseq Data

Normalization and differential analysis of expression of sRNAs previously annotated in the genome of *S*. Typhimurium SL1344 [28] were performed using DESeq2 version 1.2.5 [25] then integrated in COV2HTML [27] for data analysis with a cut off ratio of 2.

### Northern Analysis

Total RNA from *Salmonella* strains grown for 18 H in LB at 37°C was fractionated on an 8% polyacrylamide–7 M urea gel and transferred to Hybond-N+membranes (RPN1520B GE Healthcare). Blots were hybridized to DNA oligonucleotides (Table S2) labeled at the 5′ends with T4 polynucleotide kinase using the UltraHyb-OLIGO buffer (AM8663, Ambion).

### Quantitative Real-time PCR

Quantitative real-time PCR was performed to verify the transcriptomic data using Applied Biosystems 7300 Real-Time PCR system. Total RNA was extracted from cells grown to stationary phase in LB as described above. The RNA (1 µg) was reverse-transcribed 2 hours at 37°C in 50 µl of reverse transcriptase buffer in the presence of 2 mM dNTPs, 1 µl of random hexamers (1 µg/µl p(dN)_6_, Roche) and 10 U of avian myeloblastosis virus (AMV) reverse transcriptase (Promega) and 40 U of recombinant ribonuclease inhibitor (Rnasin, Promega). The relative amounts of target mRNA were determined by real-time PCR using the Fast Start Universal SYBR Green Master following the manufacturer’s instructions (Roche). A final dissociation curve analysis step from 60°C to 95°C was performed to confirm the amplification specificity. To check whether contaminating chromosomal DNA was present, each sample was tested in control reactions that did not contain reverse transcriptase. The real-time PCR was performed using 200 nM gene-specific primer pairs (Table S2) designed *in silico* using Primer3 software (http://primer3.ut.ee) to generate amplicons in the 100–150 bp range. A relative standard curve experiment using a ten-fold dilution series of genomic DNA was performed for each primer pair to determine the amplification efficiency. The efficiency of the amplification for all the genes tested was higher than 1.8. Three biological replicates were analysed in duplicate each. *rpoZ* was used as reference gene as it displays little variation in the transcriptional studies performed in our lab using wild-type and Δ*rpoS* strains. Gene expression levels were calculated using the comparative Ct method (2^−ΔΔCT^) as previously described [29]. P values were calculated using a two-tailed t test.

### Enzymatic Assays

β-galactosidase activity was measured as described by Miller [30] and is expressed in Miller units.

### Sequence Analyses

DNA and amino acid sequence analyses were conducted using the BLAST programs at the NCBI (National Center for Biotechnology Information). Functional annotations were obtained from the MicroScope Microbial Genome Annotation & Analysis Platform (www.genoscope.cns.fr/agc/microscope/home/index.php) [31] and the KEGG server (www.genome.jp/kegg/kegg2.html). Functional analysis of genes with significant changes in expression was done using clusters of orthologous groups (COG) functional categories described for *Salmonella enterica* serovar Typhimurium ATCC14028 genes (www.genoscope.cns.fr/agc/microscope/genomic/classifCOG.php).

### Motility Assay

Three independent stationary phase cultures of strains grown in LB (18 h, 37°C, 200 rpm) were used. 1 µl of culture was inoculated into 0.3% agar LB plates that were incubated at 37°C for 5 h.

### Competition Assays

Overnight LB cultures were washed and resuspended in phosphate- buffered saline (NaCl 137 mM, KCl 2.7 mM, Na_2_HPO_4_ 10 mM, KH_2_PO_4_ 1.76 mM) to an OD600 of 1.0. Equal numbers of cells of the wild-type strain ATCC14028 and the mutant strain were then mixed in fresh LB medium to give a total of about 3000 cells ml-1 and the mixture was incubated at 37°C with shaking. Aliquots of bacteria were removed at timed intervals and numbers of viable cells of each strain were determined on LB plates containing the appropriate antibiotics. P values were calculated using a two-tailed t test.

## Results and Discussion

### Global Gene Expression in Wild-type and Δ*rpoS Salmonella* Strains

**Figure 1:**
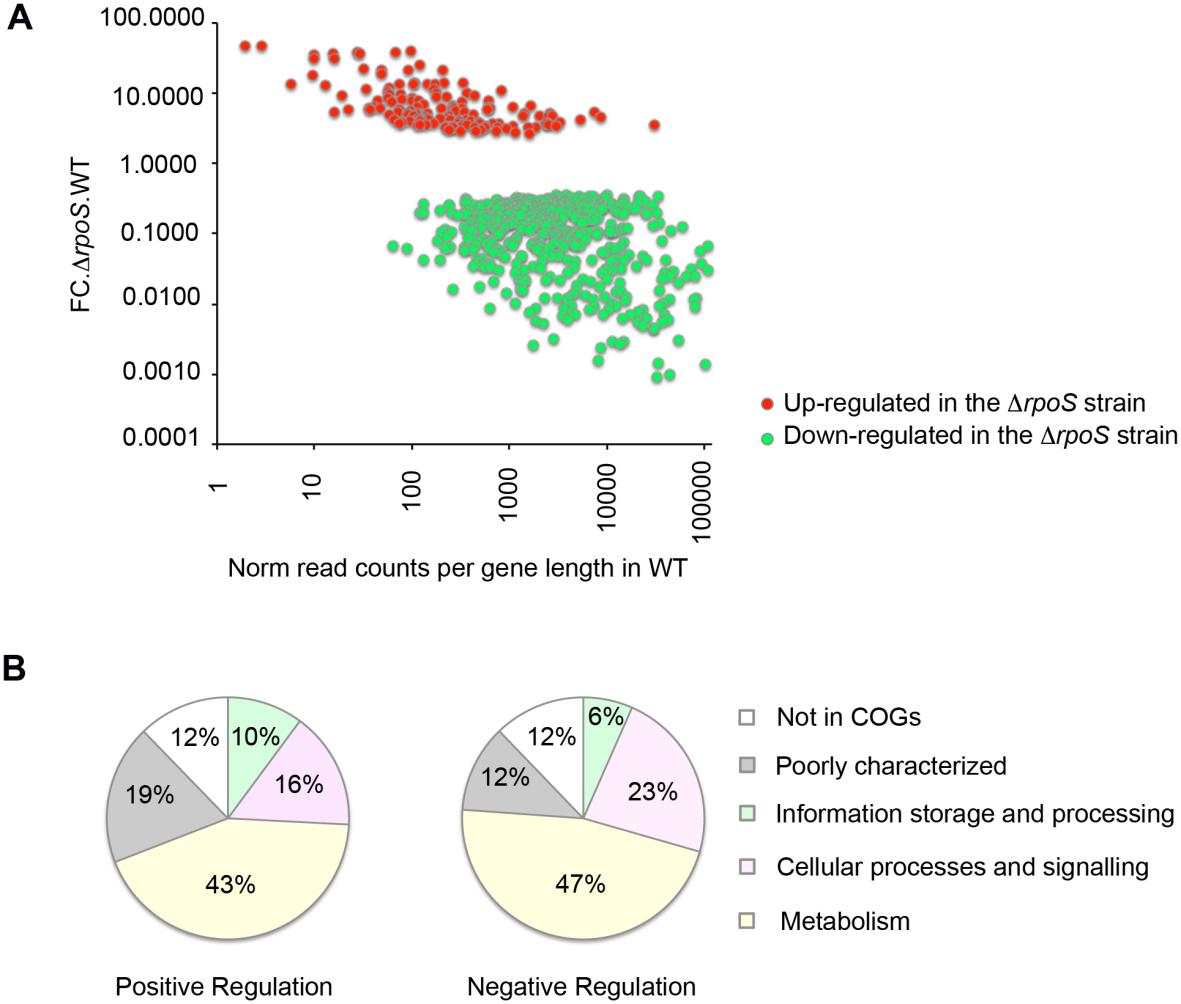
Directional RNAseq data analyses. (A) Relative expression level and σ^S^-dependency of σ^S^-dependent genes (p<0.001). The x axis shows reads counts in the wild-type strain VF7969 normalized to the lenght of the gene. The y axis shows the fold change in the expression levels of the gene in the Δ*rpoS* strain VF9356 compared to the wild-type strain (as reported in Dataset S2). Red and green dots represent genes negatively and positively controlled by σ^S^ respectively. (B) Functional categories of σ^S^-controlled genes (p<0.001). Genes controlled by σ^S^ are grouped according to their functional categories in the COG database (detailed COG assignments are given in Dataset S2). The relative occurrence of genes belonging to each category in the set of genes positively controlled by σ^S^ (left pie chart) and negatively controlled by σ^S^ (right pie chart) is shown. Some of the genes do not currently have a COG functional category assignment (here represented as not in COGs). Note that some genes have multiple COG category assignments (Dataset S2).

To assess the relative impact of σ^S^ at a global level, transcript levels of wild-type and Δ*rpoS* strains of *Salmonella* were measured by directional RNA-sequencing using three biological replicates of strains grown to stationary phase in LB (GEO GSE46380). σ^S^ is known to accumulate during entry to stationary phase in rich medium and to reach its maximum level of production in late stationary phase [14]. Consistently, in a previous study using RNA sequencing and chromatin immunoprecipitation methods to evaluate transcription in *S*. Typhimurium in rich medium, σ^70^ was the main σ factor at early stationary phase [28], suggesting that most σ^S^-regulated genes are expressed in late stationary phase. We thus isolated total RNA from cells in late stationary phase. We identified a total of 1071 genes differentially expressed in the wild-type strain and the Δ*rpoS* mutant (p<0.05), of which 607 were highly significant (p<0.001) (Dataset S1 and Figure 1). In general, genes up-regulated in the Δ*rpoS* mutant (145 genes, p<0.001) exhibited lower expression levels and fold-change values than down-regulated genes (462 genes, p<0.001) (Figure 1A). Some σ^S^-dependent genes are likely directly regulated through binding of σ^S^ to promoters while others are likely regulated indirectly by σ^S^.

### Physiological Functions of the σ^S^ Network in *S*. Typhimurium

Among ATCC14028 genes that have been assigned to a category of orthologous genes (COG), the most prominent categories associated with σ^S^ regulation were metabolism, transcription, signal transduction mechanisms and membrane biogenesis and unknown functions (see detailed COG assignments in Dataset S2 and an overview in Figure 1B).

**Figure 2:**
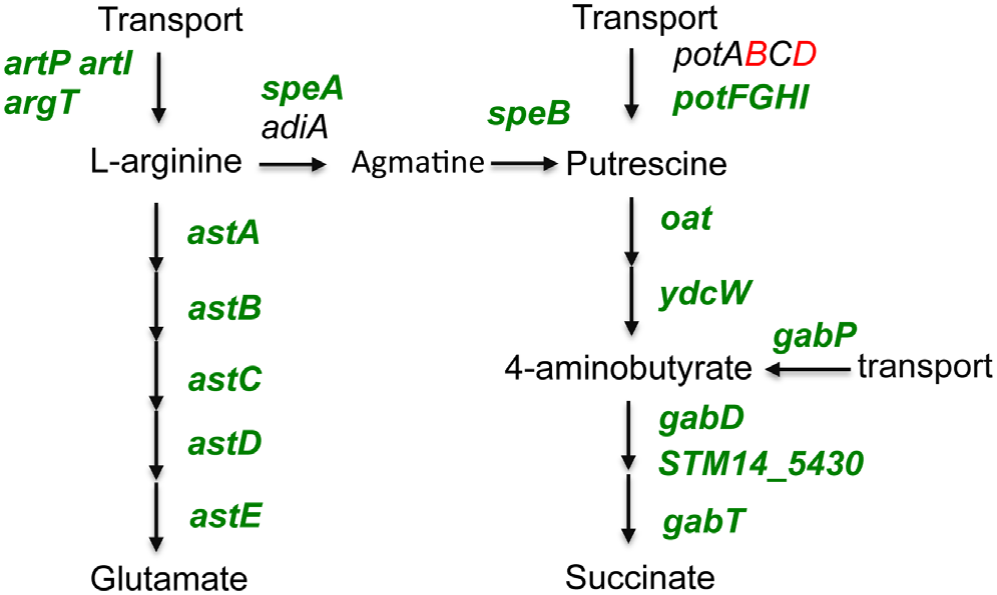
Role of σ^S^ in the transport and catabolism of L-arginine. Schematic representation of σ^S^-dependent pathways involved in metabolism of L-Arginine, putrescine and 4-aminobutyrate. To assess the contribution of σ^S^ in the expression of the metabolic pathways indicated, genes differentially expressed with a p value of less than 0.05 in the wild-type and Δ*rpoS* strains of *Salmonella* were considered (Dataset S2). Genes showing differential expression with p<0.001 are indicated in bold face. Genes in red and green were negatively and positively controlled by σ^S^ respectively. Genes in black did not show differential expression in the wild-type and Δ*rpoS* strains.

σ^S^ had a substantial effect on expression of metabolic genes, primarily for energy production/conversion and transport/metabolism of carbohydrates, amino acids and inorganic ions (Dataset S2). In some cases, most genes in a given pathway are controlled by σ^S^, suggesting a role for this pathway in stationary phase physiology (see for instance pathways shown schematically in Figure 2 and Figures S1–S2, with genes activated and down-regulated by σ^S^ in green and red, respectively). A number of genes involved in central energy metabolism exhibited positive σ^S^ control (phosphotransferase systems, glycolysis, the pentose phosphate pathway, mixed acid fermentation, and acetate metabolism) whereas genes encoding enzymes in the tricarboxylic acid (TCA) cycle and the operons encoding NADH dehydrogenase-I (*nuo*) and ATP synthase (*atp*) were down-regulated by σ^S^ (Dataset S2 and Figure S1). σ^S^ might thus play a role in transition from aerobic respiration towards more fermentative and/or anaerobic respiratory energy metabolism in stationary phase *Salmonella*.

Since σ^S^ controls, either positively or negatively, a large number of genes, and it is required for complex phenotypes such as multiple stress resistance and biofilm formation, it is difficult to pinpoint specific genes directly involved in a particular physiological function of σ^S^. However, it is likely that several σ^S^-controlled genes contribute to prevent or repair oxidative damage. First, σ^S^ controls antioxydant pathways involving catalases, superoxide dismutase, glutaredoxins and glutathione-S-transferases (Dataset S2). In addition, σ^S^ activates genes encoding ferritins/ferrochelatase and Fe-S repair proteins (including *dps, bfr, hemH, sufABCDSE*, Dataset S2) and controls genes coding for manganese/iron acquisition functions (including activation of *mntH, sitABCD, iroBCN* and down-regulation of *feoB, fepEC,* Dataset S2). These findings suggest that σ^S^ controls iron use and the concentration of free iron in the cell. This strategy would be consistent with the role of σ^S^ in preventing oxidative stress since iron can promote the formation of reactive oxygen species (ROS) during aerobic metabolism [32]. In addition to activating the *suf* genes encoding an alternative system for iron-sulfur clusters assembly, σ^S^ down-regulated the *hscBA* genes (Dataset S2) encoding chaperones with central roles in assembly of iron-sulfur clusters mediated by the housekeeping Isc system [33]. This suggests that stationary phase cells relay upon the alternative Suf machinery, rather than the housekeeping Isc system, for Fe-S cluster assembly, an hypothesis consistent with the findings that Suf is more resistant to oxidation than Isc and is functional under iron-limiting conditions [33].

σ^S^ appears to trigger switching between certain isozymes (see differential regulation of *talA/talB, tktA/tktB, acnA/acnB, pykF/pykA, fumA/fumB/fumB*, *sodA/sodB* and *nrdAB/nrdEF* Figure S1 and Dataset S2). σ^S^ can modulate expression of isoenzymes more functional during stationary phase and/or that display key features increasing their activity or stability in these conditions. For instance, selection of isozymes resistant to oxidation and iron depletion [34] and replacement of iron-containing enzymes with iron-independent isoenzymes [35] may be key characteristics of the stationary phase physiology, consistent with the effect of σ^S^ in metal acquisition function mentioned above.

σ^S^ controlled carbon storage genes for production and degradation of glycogen and the osmoprotectant trehalose (Figure S2) [3] and seems to play an important role in the transport and utilization of amino acids such as L-arginine (Figure 2). The LB medium is rich in amino acids [36] and arginine is a nitrogen reservoir and the precursor for polyamine biosynthesis (Figure 2). These small cationic amines are involved in a variety of functions including resistance to oxidative stress and antibiotics, stabilisation and condensation of DNA during senescence, RNA and protein synthesis and virulence [37]. σ^S^ activated genes for transport, synthesis and degradation of putrescine in *Salmonella* (Figure 2) and *E. coli* K-12 [6] and might control its intracellular concentration.

Some genes up-regulated in the Δ*rpoS* strain are probably induced in response to cellular damages derived by the lack of a functional σ^S^ protein. For instance, the heat shock protein encoding genes *ibpAB* and *groEL/groES* were up-regulated in the Δ*rpoS* strain possibly because the level of damaged proteins increased in the absence of σ^S^. Indeed, the levels of carbonylated proteins increased in stationary phase *E. coli* strains lacking σ^S^ likely as a result of increased endogeneous oxidative stress [38]. In addition, whereas the σ^S^- mediated induction of genes encoding proteases (including *htrA, clpX, ptrB, yggB, tldD, hslV*, Dataset S2) may favor the recycling in stationary phase of mis-folded proteins as nutrients, a low level of expression of these genes in the Δ*rpoS* mutant may contribute to accumulation of damaged proteins.

**Figure 3:**
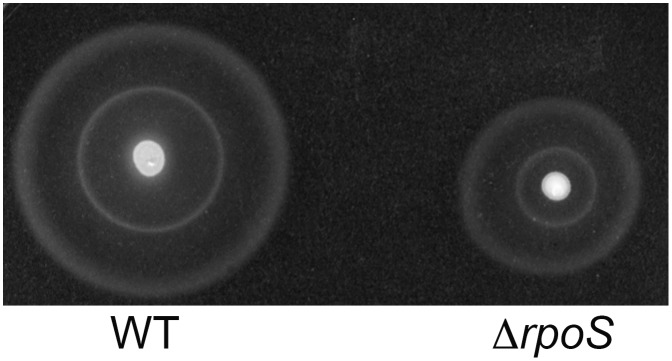
Motility of *Salmonella*Δ*rpoS* mutant. Motility on 0.3% agar LB plates of the wild-type strain ATCC14028 (WT) and its Δ*rpoS* mutant VFC331 after 5 h at 37°C.

Altogether, these data on the σ^S^-dependent *Salmonella* transcription are in general agreement with previous studies in *E. coli* K-12 [3], [6]–[9]. One major exception concerns the expression of genes associated with motility. Genes encoding the flagellar sigma factor FliA, flagellar proteins and motor components are down-regulated by σ^S^ in *E. coli* K-12 [3], [7]. In contrast, the flagellin genes *fliC*, and to a lesser extent *fljB*, were positively controlled by σ^S^, even though transcription of the *flhDC* genes encoding the master regulator of flagellar synthesis was slightly up-regulated in the *Salmonella* Δ*rpoS* strain. In addition, the *Salmonella* Δ*rpoS* mutant showed a decrease in motility compared to the wild-type strain (Dataset S2, Figure 3). Given the complexity of regulatory controls affecting motility [3], it is not clear whether the positive regulation of *fliC* accounts for the effect of σ^S^ on motility or whether σ^S^ acts through other ways as well. σ^S^ also positively regulated flagellar gene expression in another strain of *S.* Typhimurium, SL1344, and in other pathogens such as *Vibrio, Legionella* and *Pseudomonas* [39].

### Negative Regulation by σ^S^ and Bacterial Fitness

Whereas σ^S^ has a positive effect on a large number of genes that likely contribute to stress resistance, it has also a negative effect on the expression of several genes under the control of other sigma factors (Dataset S2) [3], [7], [12], [40]. These negative effects of σ^S^ on gene expression likely drive the selection of non-functional *rpoS* alleles in environments with no stress, where reduced σ^S^ activity confers a growth advantage [3], . These observations have led to the proposal that the acquisition of stress resistance mediated by σ^S^ comes at the expense of growth capabilities as a consequence of a regulatory antagonism between σ^S^ and other σ, mainly σ^70^ [3], [41], [42]. The implications of negative regulation by σ^S^ in the stationary phase physiology have not been studied in detail.

We believe that an important issue to be addressed in future experiments is whether and how the negative effects of σ^S^ on gene expression confer any fitness advantage to the bacteria. Genes down-regulated by σ^S^ may show antagonistic phenotypic pleiotropy (*i.e.* their expression is advantageous in some environmental conditions and detrimental in others). Optimizing bacterial fitness in a defined constant environment would require selection for and against these genes, and this evolutionary force would drive gene loss or inactivation, in line with the selection of *rpoS* mutants in some environmental conditions [11], [12]. However, in fluctuating environments, fine-tuning regulatory processes by σ^S^ might be used to adapt bacterial fitness to a variety of natural habitats, including host niches.

Negative effects of σ^S^ on the respiratory chain might contribute to the antioxidant defenses by reducing the production of ROS as toxic by-products of aerobic metabolism [3], [32] and might redirect NADH usage to fuel the activity of antioxidant enzymes. Indeed, accumulation of NADH following the inhibition of *Salmonella*’s electron transport chain by nitric oxide has been identified as an antioxidant strategy [43]. Interestingly also, inhibition of ATP synthase-promoted proton translocation and ATP synthesis is a strategy utilized by *Salmonella* during infection to control ATP levels and maintain physiological cytoplasmic pH, and membrane potential [44]. Therefore, σ^S^-mediated reduction in the synthesis of the ATP synthase might enable *Salmonella* to maintain a physiological cytosolic pH and modulate its membrane potential for optimal survival under starvation conditions. Furthermore, down-regulation by σ^S^ of the respiratory complexes I (NADH dehydrogenase Nuo) and II (succinate dehydrogenase Sdh) and the σ^S^-dependent switch from the Isc to the Suf Fe-S cluster biosynthesis machinery might reduce the uptake of antibiotics. Indeed, it has been recently shown that, during iron limitation, *E. coli* cells become intrinsically resistant to aminoglycosides by switching the Fe-S cluster biosynthesis machinery from Isc to Suf and down-regulating respiratory complexes I and II [45]. The Suf system cannot efficiently mature these respiratory complexes, resulting in impairment of the proton motive force, which is required for bactericidal aminoglycoside uptake [45].

Besides effects of σ^S^ in metabolism and the respiratory chain functions, additional negative effects of σ^S^ on gene expression might contribute to bacterial fitness. σ^S^ controls mutagenesis induced by subinhibitory concentrations of antibiotics *via* the down-regulation of *mutS*, a gene involved in mismatch-repair [46]. It is tempting to speculate that negative regulation of *mutS* expression by σ^S^ (Dataset S2) might contribute to the appearance of antibiotic resistant mutants, and consequently the survival of bacterial populations in environments containing antibiotics. Down-regulation of porins (for example encoded by *ompC, ompF, ompD/nmpC* and *ompW*, Dataset S2) might also confer resistance to antibiotics and other toxic compounds and bacteriophages [47]. More generally, σ^S^ controls genes encoding membrane proteins and transporters, especially those belonging to the ATP-Binding Cassette transporter family, suggesting altered membrane composition and traffic in stationary phase (Dataset S2). This membrane remodeling may be directed towards nutrients scavenging and increased resistance against toxic compounds and physical assaults, an hypothesis consistent with the observed positive effect of σ^S^ in cell envelope resilience in *E. coli* [48]. Negative control by σ^S^ of surface determinants that are targets for a protective antibody response, such as OmpD [49], may also contribute in the escape of immune response during host infection.

### Hierarchical Regulation and Regulatory Loops in the σ^S^-Network and Interplay with other Global Regulators

Because the number of sigma factors exceeds that of the core RNAP, sigma factors compete for binding to the core RNAP available in the cell [2], [3]. Many genes down-regulated by σ^S^ (Dataset S2) are transcribed in *Salmonella* from promoters showing characteristics of σ^70^-dependent promoters [28], [50]. Negative control by σ^S^ is likely in part an indirect effect. According to the current model of negative regulation by σ^S^ invoking σ competition [3], [40], [42], σ^70^-dependent genes are up-regulated in the absence of σ^S^ because they are expressed from promoters that are sensitive to the increase in the cellular concentration of Eσ^70^ that might result from a lack of competition between σ^S^ and σ^70^ for E binding. However, global regulation by σ^S^ may also involve intermediate regulators in the σ^S^ network, including repressor molecules.

**Figure 4:**
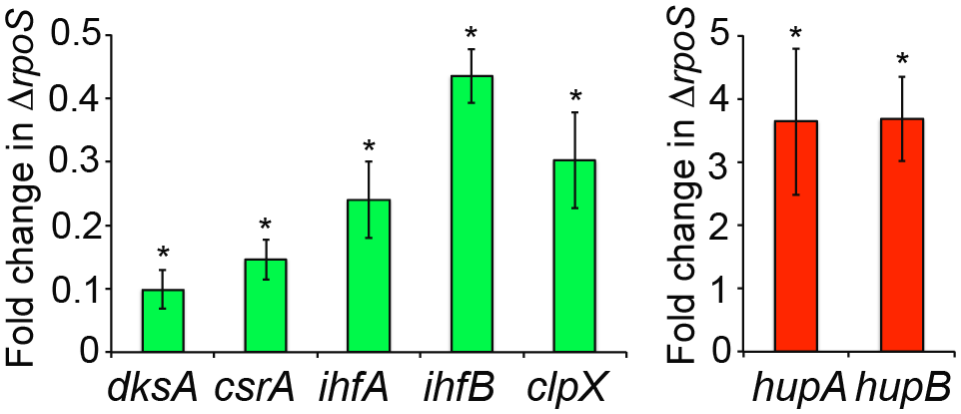
σ^S^-dependent transcriptomic expression of pleitropic regulators. Verification of the σ^S^-dependent transcriptomic expression of *csrA, dksA, ihfA, ihfB, hupA, hupB* and *clpX* by quantitative RT-PCR. RNA extracted from stationary phase LB cultures (18 h) of *Salmonella* wild-type and Δ*rpoS* strains (VF7969 and VF9356) was reverse transcribed to cDNA and used as a template for qRT-PCR. *rpoZ* was used for normalization. Red and green bars correspond to genes negatively and positively controlled by σ^S^, respectively. Three biological replicates were analysed in duplicate each and error bars display the standard error of the mean. *, expression levels in the Δ*rpoS* mutant significantly different from that in the wild-type strain (p<0.05).

As a first step to explore indirect regulation by σ^S^, the possible regulatory functions of the σ^S^-controlled downstream network were examined. σ^S^ affected the transcript levels of numerous genes encoding known or putative signal transducing and/or DNA-binding proteins (Figure 1B and COGs T and K, Dataset S2), suggesting that σ^S^ controls the transcription of many secondary transcription factors. Genes for global regulators (*csrA, soxS, arcA* and to a lesser extent *ompR*), abundant nucleoid-associated proteins (*ihfAB, cbpA, hupAB* and to a lesser extent *stpA*), and modulators of RNAP activity (*dksA* and to a lesser extent *greA, nusG*) appeared differentially expressed in the wild-type and Δ*rpoS* strains (Dataset S2). σ^S^-dependent transcription of *cbpA*, *ifhAB* and *csrA* has been reported in *E. coli* K-12 [51]–[54] and *arcA* appeared slightly down-regulated in a *E. coli* Δ*rpoS* mutant [8]. Consistent with the RNA-seq data, quantitative reverse transcriptase-polymerase chain reaction showed that transcription of the regulatory genes *csrA, dksA, ihfA* and *ihfB* is positively controlled by σ^S^ whereas the *hupA* and *hupB* genes are down-regulated by σ^S^ (Figure 4, Dataset S2). Also, the *clpX* gene, encoding a subunit of the ATP-dependent complex ClpXP protease, involved in proteolysis of many proteins including σ^S^ [2], [3] is activated by σ^S^ (Figure 4, Dataset S2). Although these data showed that transcripts levels for *csrA, dksA, ihfAB, hupAB* and *clpX* are modulated by σ^S^ in late stationary phase in *Salmonella*, additional experiments are required to determine whether wild-type and Δ*rpoS* cells differ in the global activity of the corresponding gene products. Indeed, other factors capable of differentially influencing protein levels and activity of these regulators might compensate for the observed variations in their transcript levels in the absence of σ^S^.

**Figure 5:**
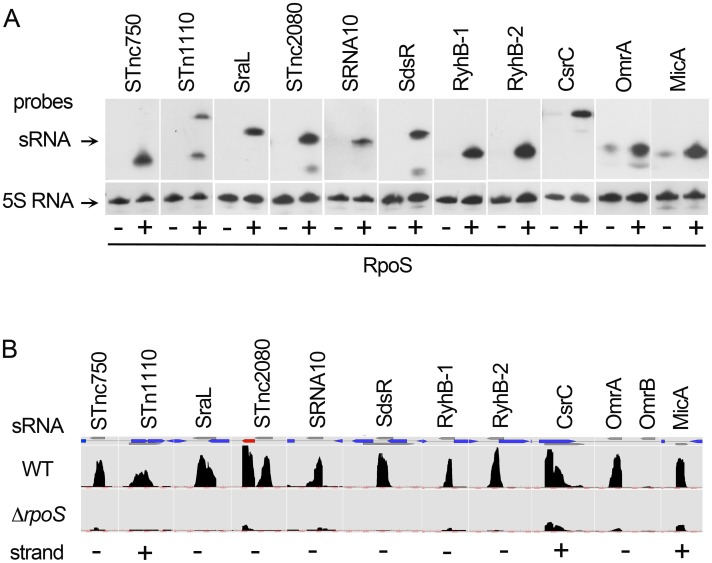
σ^S^-dependent expression of small RNAs in *Salmonella*. (A) The indicated sRNAs were detected in Northern experiments in the wild-type strain ATCC14028 (+) and its Δ*rpoS* derivative VFC331 (−). The positions of bands were in agreement with the expected transcript lengths [28], [64], [65] (Table S3) except for STnc1110 (195 nt, detected with an additional band of 120 nt) that might be processed in late stationary phase. Blots were stripped and re-probed with 5S RNA probe to confirm loading of equal quantities of wild type and Δ*rpoS* RNA. (B) Mapped reads, in the wild-type and Δ*rpoS* strains, VF7969 and VF9356 respectively, of the σ^S^-dependent RNAs assessed in Northern experiments. The mapped reads were formatted into graph files for visualization at a strand-specific manner using COV2HTML. The annotated sRNA genes are indicated as grey arrows and open-reading frames annotated in ATCC14028 are shown as blue arrows. Open-reading frames overlapping the sRNAs are small putative CDS of unknown function annotated in ATCC14028. STnc2080 is located upstream of STM14_3200, indicated in red, a gene directing the synthesis of a tRNA-Arg and activated by σ^S^ (Dataset S2). SdsR is complementary to a portion of the sRNA transcribed from the opposite strand, SraC. The scale for read counts on the y axis was 1–250 for STnc1110 and sRNA10, 1–1000 for OmrA and MicA, 1–2500 for STnc750 and RyhB-1, 1–5000 for STnc2080 and RyhB-2, 1–10000 for SraL, SdsR and CsrC.

As an example, interesting regulatory antagonisms were observed. *csrA,* encoding a post-transcriptional global regulator, and the small RNA CsrC are both positively controlled by σ^S^ (Dataset S2, Figures 4–5 and see paragraph below). CsrA acts mostly negatively by binding and destabilizing mRNAs [53]–[56]. CsrC binds and sequesters CsrA, thereby inhibiting its activity [53], [57]. The other components of the Csr system include the sRNA CsrB, that also binds and sequesters CsrA, and CsrD, a protein that participates in degradation of CsrC and CsrB [53], [56]. In the conditions used, *csrB* and *csrD* were detected to low and similar levels in wild-type and Δ*rpoS* strains (data not shown). The regulation of expression of the Csr system is complex [53], [54]. CsrA indirectly activates its own transcription while repressing its own translation and also controls production of CsrC/CsrB/CsrD. Transcription of *csrA* in *E. coli* is controlled by several promoters, two of which are σ^S^-dependent [54]. It is conceivable that σ^S^ modulates the fine-tuned balance of the Csr system and uses this system to indirectly regulate target genes at the post-transcriptional level. Also, transcript levels for σ^E^ and for its antisigma factor RseA and its coantisigma factor RseB, encoded by the same operon, are all reduced in the Δ*rpoS* strain, compared to the wild-type (3.5 fold p<0.001 for *rseA* and about 2 fold p<0.05 for *rpoE* and *rseB*, Dataset S2). Since σ^E^ has a positive effect on σ^S^ expression in stationary phase [10], a possible control of σ^E^ expression and/or activity by σ^S^ would not be unexpected. σ^S^ may have several self-regulatory circuits by controlling the expression of numerous genes that modulate its expression [2], [3], for instance, *clpX, rssB/hnr, arcA, hupAB, dksA,* and *dsrA* (p<0.001, Dataset S2). Since these regulators may work either cooperatively or independently, or even have opposing effects on σ^S^ expression [2], [3], σ^S^ self regulatory control may be important for maintaining a proper level of σ^S^ and for integration of signal inputs. Future experiments will assess whether these changes in expression of global regulators at the transcriptome level are transferred at the functional level and whether some of the σ^S^-controlled secondary regulators are intermediate regulators in regulatory cascades and/or contribute to Eσ^S^-mediated regulation in feedforward regulatory loops.

Besides the control of regulatory proteins, control of metabolic/signaling enzymes by σ^S^ might lead to variations in levels of signalling molecules and affect protein modifications and/or gene expression at different levels. For instance, genes involved in the metabolism of the second messenger C-di-GMP [3], [55], [58] showed differential transcript levels in the wild-type and Δ*rpoS* strains (*yaiC, ydiV, yegE*, STM14_2408, STM14_2209, STM14_5555, STM14_4086, STM14_2047, Dataset S2). Also, putrescine affects global gene expression [37] and the control of its intracellular levels (Figure 2) might be a mechanism of indirect gene regulation by σ^S^. Since σ^S^ plays a central role in metabolism, it may affect the levels of intermediate metabolites with signalling functions such as CoA derivatives, NADH/NADPH, glutamate, acetate and acetyl-phosphate.

### σ^S^-dependent sRNAs

sRNAs are important pleiotropic regulatory elements [59]–[61]. Some sRNAs are positive regulators but the majority of sRNAs negatively regulate their targets by translational repression and/or destabilization of the mRNA [59]–[61]. Thus, sRNAs might be important contributors to negative regulation of gene expression by σ^S^, as recently shown for σ^E^ [62]. More than one hundred sRNAs have been recently annotated in *S*. Typhimurium SL1344 [28]. Corresponding coordinates of these sRNAs in the genome of ATCC14028 are listed in Table S3. The RNA-seq data offered the possibility to assess whether some of these annotated sRNAs are differentially expressed in the wild-type strain and Δ*rpoS* mutant. Fourty-two sRNA showed differential expression levels (>2 fold, p<0.001) in the wild-type and Δ*rpoS* strains (Table 1) and the σ^S^-dependent control of eleven of them was confirmed by Northern experiments (Figure 5). Of these, only four (SraL, SdsR and RyhB-1/-2) were known to be controlled by σ^S^ [63]–[65] whereas the other sRNAs are novel σ^S^-targets. Some sRNAs differentially expressed in wild-type and Δ*rpoS* strains were detected to low levels in the growth condition used (Table 1). Control of their expression by σ^S^ might be amplified or abolished in other growth conditions. Indeed, the σ^S^ control of gene expression depends on growth conditions and many genes are fully expressed under the control of σ^S^ under a specific condition [3], [8].

**Table 1:** sRNAs differentially expressed in wild-type and Δ*rpoS* strains.

sRNA	Mean *rpoS*	Mean WT	Fold Change^a^	Start	End	Strand
CsrC	4333	19747	0.26	4223741	4223984	+
CyaR	345	56	4.99	2282684	2282769	+
DsrA	694	217	2.83	2080053	2080139	−
GcvB	2	17	0.24	3155126	3155326	+
GlmY	7111	17770	0.43	2759217	2759400	−
IsrI	7	248	0.05	2812865	2813112	−
IstR-1,2	67	283	0.29	4011708	4011839	−
MicA	99	494	0.24	2987088	2987161	+
OmrA	28	621	0.07	3189931	3190017	−
OxyS	9	41	0.29	4356452	4356570	−
RybA	104	334	0.36	903092	903188	−
RybB	59	229	0.3	943606	943684	−
RybD	90	10	5.26	808431	808515	+
RydC	2789	402	5.33	1739650	1739715	+
RyeF	72	419	0.21	2012350	2012656	−
RygC	466	2565	0.22	3242088	3242232	+
RygD	913	3695	0.28	3380548	3380692	−
RyhB-1	124	1238	0.14	3729100	3729194	−
RyhB-2	165	4480	0.06	1362850	1362950	−
SdsR	3	4823	<0.001	1979458	1979560	−
SraC	122	17	5.37	1979380	1979690	+
SraL	38	23180	<0.001	4518412	4518552	−
sRNA10	6	117	0.09	680323	680422	−
SroC	13056	49251	0.32	729258	729410	−
STnc1060	32	109	0.35	467925	467990	−
STnc1080	133	42	2.76	1064537	1064598	−
STnc1110	13	204	0.1	1696589	1696782	+
STnc1200	12	2	3.79	926560	926629	−
STnc1220	1	9	0.26	1501842	1501914	−
STnc1280	763	290	2.41	2093821	2093893	+
STnc1300	373	129	2.57	2125107	2125245	+
STnc1330	6	1246	0.01	2322111	2322325	+
STnc1380	84	14	4.47	2783476	2783543	−
STnc1390	41	137	0.35	1294132	1294195	−
STnc150	4	30	0.2	1335643	1335799	−
STnc1560	97	312	0.36	2502911	2503019	+
STnc2080	45	3081	0.03	2813257	2813365	−
STnc290	144	26	3.81	3214058	3214136	−
STnc540	46	181	0.3	1429394	1429487	+
STnc570	461	2600	0.22	1603700	1604389	−
STnc580	13	1	4.66	1759908	1760029	−
STnc750	36	1258	0.05	3259604	3259692	−

a Fold change estimated by DESeq2 using normalized means.

(p<0.001).

The σ^S^-control of sRNAs might be direct (for instance in the case of SdsR and SraL) [63], [64] or indirect. In particular, basal expression levels in stationary phase of some sRNAs such as OmrA and MicA were detected in the Δ*rpoS* mutant suggesting the existence of alternative mechanisms of expression. Some of the σ^S^-dependent sRNAs are regulated by other regulators such as Fur, σ^E^ and OmpR [59]–[61], and regulatory interactions linking these regulators and σ^S^ in stationary phase might result in the control of the sRNA by σ^S^. In contrast to OmrA, the highly-similar and OmpR-regulated sRNA OmrB [28], [59] was detected in very low levels in the conditions used in this study (Figure 5B), suggesting that these two sRNAs are differentially regulated in late stationary phase.

Since sRNAs act by direct pairing with multiple mRNA targets [59]–[61], they might have pleiotropic effects and significantly expand the regulatory role of σ^S^ at the post transcriptional level. The targets of most of these sRNA are unknown so far but some of them have pleiotropic effects in outer membrane protein synthesis, metabolism remodeling, motility and biofilm formation [28], [59]–[61]. For instance, RyhB-1/RyhB-2, OmrA, MicA and SdsR down-regulate expression of many genes [59]–[61], [64]–[68] including genes negatively controlled by σ^S^ (*sodB, sdh, acnB, ompD, mutS,* Dataset S2). In *E. coli* RyhB inhibits the production of iron-storage and iron-using proteins during growth under iron-limiting conditions and it has been proposed that this regulation enables iron sparing for essential pathways [68]. Experiments are underway to assess to which extent RyhB sRNAs might play a role in σ^S^-dependent modulation of iron use in late stationary phase. During the preparation of this manuscript, SraL was shown to be controlled by σ^S^ and to down-regulate the expression of a chaperone encoded by the *tig* gene and involved in protein folding [63]. Under the conditions used in our study, the σ^S^-dependent control of SraL (Figure 5) did not significantly affect *tig* transcripts levels or this effect was masked by compensatory regulations in the network. A few sRNAs appeared down-regulated by σ^S^ (Table 1). Among those displaying the highest fold change in their expression levels between wild-type and Δ*rpoS* strains, RydC activates translation of the *cfa* mRNA produced from a σ^70^-dependent promoter [69]. *cfa* encodes a cyclopropane fatty acid synthase which modifies phospholipids and contributes to the stability of the bacterial membrane and acid resistance [3], [69]. In stationary phase, σ^S^ activates expression of the *cfa* gene (Dataset S2) [3] and the σ^S^-dependent promoter yields a shorter isoform of the *cfa* mRNA, insensitive to RydC regulation [69]. Thus, in stationary phase, when *cfa* transcription relies on σ^S^, RydC might be dispensible and possibly detrimental for expression of other stationary phase genes and its expression might be downregulated accordingly by σ^S^. Alternatively, RydC might be up-regulated in the Δ*rpoS* strain to activate synthesis of cyclopropane fatty acid synthase and compensate for the absence of σ^S^-induction of *cfa* transcription (*i.e.* RydC-mediated activation of *cfa* might be a backup mechanism).

σ^S^-dependent sRNAs might also target proteins instead of mRNAs [59]. As mentioned above the finding that *csrC* and *csrA* were both positively controlled by σ^S^ (Figures 4–5, Dataset S2) reveals an interesting regulatory antagonism and suggests that σ^S^ modulates the fine-tuned balance of the Csr system in late stationary phase. Whereas expression of the CsrC and CsrB sRNAs is coordinated by positive transcriptional control mediated by the two-component regulatory system BarA/SirA in *Salmonella* [57], *csrC* but not *csrB* was found to be controlled by σ^S^, suggesting that these two sRNAs are differentially regulated in the stationary phase of growth.

The physiological impact of the σ^S^-dependent regulatory RNAs network and its possible connections with the hierarchical σ^S^-dependent transcriptional network will be an exciting issue for future studies.

### Inactivation of the *astA* and *sdsR* Loci has a Fitness Cost in Stationary Phase

**Figure 6:**
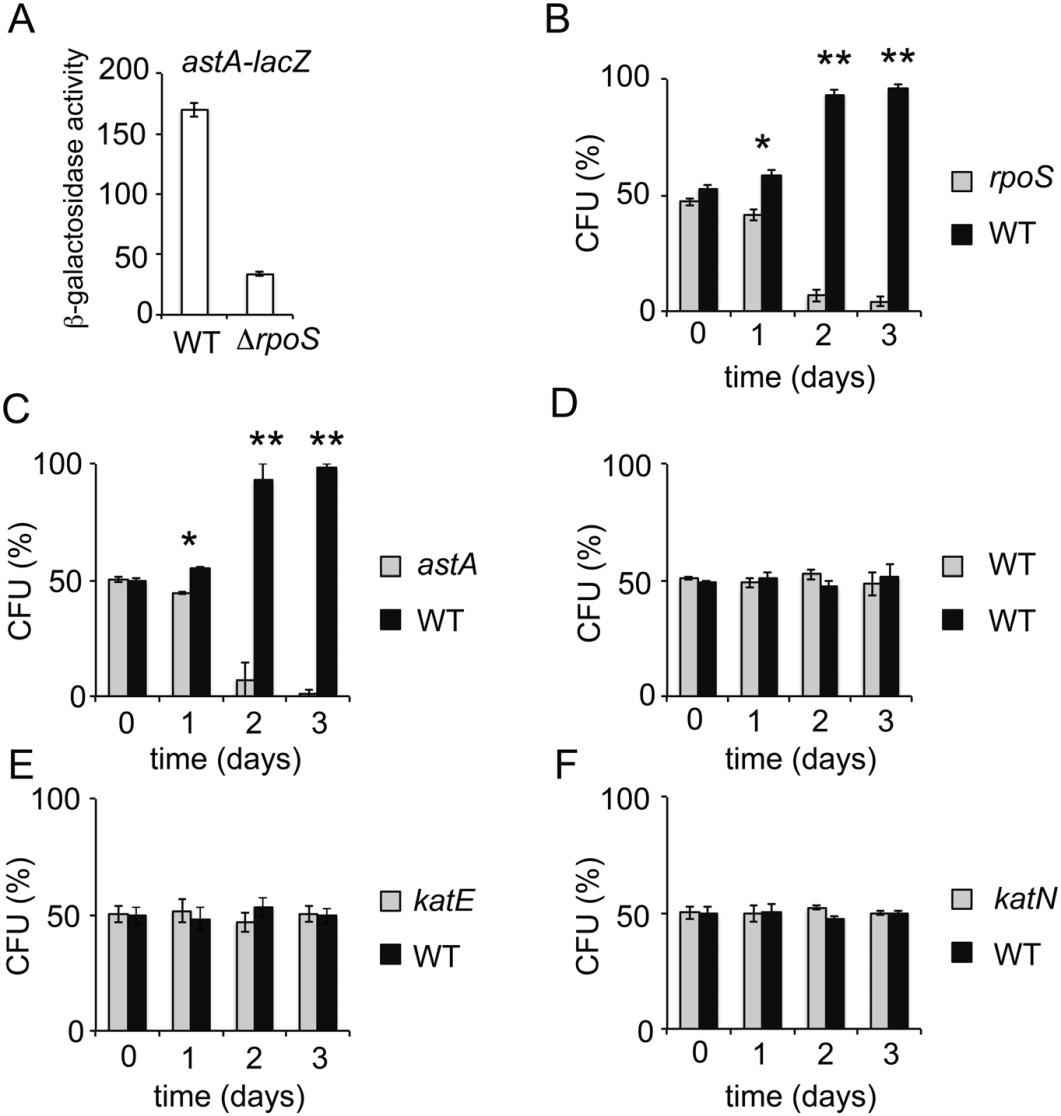
The σ^S^-dependent *ast* pathway confers a competitive fitness advantage during stationary phase. (A) Expression of a *astA-lacZ* gene fusion in *Salmonella* wild-type strain (VFD793, WT) and Δ*rpoS* derivative (VFD794) grown to late stationary phase in LB. The error bars represent standard deviations for three independent measurements. (B, C, E, F) Competition assays between the wild-type strain ATCC14028 (WT) and the mutant strain VF9356 (*rpoS*, panel B), VFD793 (*astA*, panel C), VF8082 (*katE*, panel E) and VF8088 (*katN*, panel F). (D) A control competition assay showing similar fitness of the two strains ATCC14028 and VF7969 is consistent with our previous data [14]. Equal cell numbers of the wild-type strain ATCC14028 and the mutant strain were mixed in LB medium to give a total of about 3000 cells ml-1 (time 0) and the mixtures were incubated at 37°C with shaking. Aliquots of bacteria were removed at timed intervals and numbers of viable cells of each strain were determined. Cells number of each strain is reported as a percentage of the total number of viable cells in the culture. The error bars represent standard deviations for three independent measurements. * and **, statistically significant competitive disadvantage of the mutant compared to the wild-type (*p<0.05, **p<0.0005).

Many σ^S^-dependent genes are of unknown functions (Figure 1B) and physiological roles in starved populations of most of the σ^S^-dependent genes are unexplored. Understanding what genes regulated by σ^S^ may do for the cell is an important issue for future studies. Even for genes with known functions, understanding whether and how they help bacteria deal with survival in stationary phase and stress conditions is far from being complete. Our RNAseq data pinpoint to metabolic functions as key characteristics of σ^S^ activity in *Salmonella,* as its was previously suggested in *E. coli* K-12 [3]. However, the contribution of σ^S^-regulated metabolic functions in the physiology of non/slow growing bacteria needs to be further evaluated through construction of mutations in relevant pathways. For instance, σ^S^ might activate the transport and utilization of L-arginine (Figure 2). The *astCADBE* operon required for the degradation of arginine is transcribed from two promoters, one is dependent on σ^54^, the other on σ^S^ [70], [71]. We previously isolated a mutant of *Salmonella* carrying a Tn*5*B21 transposon insertion in the *astA* gene, creating a *astA-lacZ* gene fusion [22] (Table S1). Consistent with the RNAseq data, expression of the *astA-lacZ* fusion in stationary phase was dependent on σ^S^ (Figure 6A).

We previously showed that the wild-type strain of *Salmonella* has a competitive advantage over the Δ*rpoS* mutant in stationary phase [14] (Figure 6B). To assess the impact of L-arginine degradation in maintenance metabolism, we performed similar competition experiments in which the wild-type strain and *astA* mutant were mixed in equal cell numbers in LB liquid medium and the numbers of each were followed for several days (Figure 6C). The wild-type strain ATCC14028 showed a competitive advantage during stationary phase over the *astA* mutant (Figure 6C). Three days after inoculation of the medium, more than 98% of the cells population was wild-type. In similar control experiments, the wild-type strain ATCC14028 showed similar fitness as the wild-type strain 2922 K (Figure 6D) [14]. The fitness disadvantage of the *astA* mutant was not due to the Tn5B21 insertion since strains carrying Tn*5*B21 insertions in the *katE* and *katN* genes showed similar fitness as the wild-type strain (Figure 6EF). The σ^S^-dependent *katE* and *katN* genes encode catalases [72] involved in the destruction of hydrogen peroxide (H_2_O_2_). Cellular respiration using oxygen may result in the accumulation of ROS [32]. The inactivation of catalases did not affect *Salmonella* fitness under the conditions used, possibly due to redundant functions in *Salmonella* [73]. Alternatively, as discussed above, σ^S^ may set up conditions minimizing endogenous oxidative stress and, under these conditions, catalase production might be a preventive σ^S^ response. These data showed that inactivation of the *ast* pathway has a fitness cost and thus arginine degradation may be a key feature for competitive fitness of stationary phase *Salmonella* in rich medium. Additional experiments are underway to explored the mechanistic basis for this finding.

**Figure 7:**
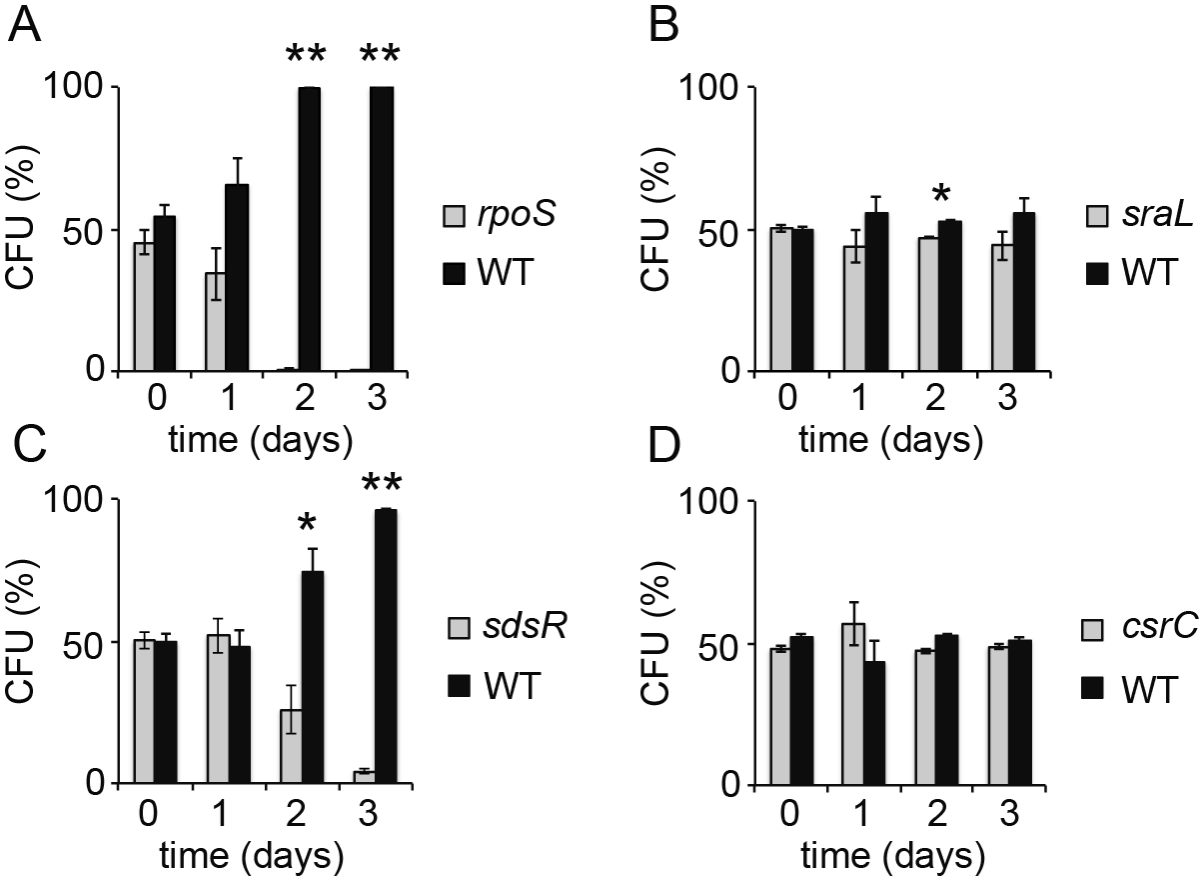
Competitive fitness of sRNA mutants during stationary phase. Competition assays between the wild-type strain ATCC14028 (WT) and the mutant strain VFC326 (*rpoS*, panel A), VFD164 (*sraL*, panel B), VFD197 (*sdsR*, panel C) and VFD510 (*csrC*, panel D). See also legend in Figure 6.

To investigate whether the competition assay could be a valuable tool to reveal the activity of sRNA in physiological conditions, we constructed *Salmonella* mutants, in which the *sdsR, sraL* and *csrC* genes were deleted and replaced by a *tetRA* cartridge, and assessed their fitness in competition experiments with the wild-type strain (Figure 7). These sRNAs were chosen because they were among the most highly expressed σ^S^-dependent sRNAs (Table 1). The *csrC* and *sraL* mutants showed similar fitness as the parental strain, while the *sdsR* mutant was outcompeted (Figure 7), indicating that deletion of the *sdsR* locus has a competitive fitness cost. Deletion of *sdsR* also inactivates the antisens overlapping gene encoding the SraC sRNA [64]. However, considering the low amount of SraC compared to SdsR (Table 1), the physiological effect of the mutation likely results from one or several of the regulatory functions of SdsR [46], [61], [64], [67] and experiments are underway to investigate this issue further. In addition, a high-throughput screening method based on the competition assay will be used to assess the impact of σ^S^-dependent sRNAs in various physiological growth conditions.

## Conclusion

This study provides insights into the positive and negative effects of σ^S^ on global gene transcription in *Salmonella* and suggests that metabolism, membrane composition, iron use and oxidative stress resistance are keys features of σ^S^ activity. This study also provides a firm basis for future studies to address molecular mechanisms of indirect regulation of gene expression by σ^S^. Our results pinpoint regulation by sRNAs as one possible mechanism mediating indirect control of gene expression by σ^S^, expanding the regulatory scope of σ^S^ at the post transcriptional level. These findings open up new fields of investigation in the regulatory network orchestrated by σ^S^ where transcriptional and post-transcriptional control mechanisms might cooperate or work in opposite direction to allow for dynamic and flexible regulatory patterns and additional signal inputs. In particular, molecular mechanisms underlying negative effects of σ^S^ on gene expression are not well documented and call for further investigation. Some of the regulatory proteins and small RNAs identified in this study might endow σ^S^ with repressor functions. The possibility that negative effects of σ^S^ on gene expression confer fitness advantage to the bacteria is also an interesting issue for future studies. Down-regulation of expression by σ^S^ might target genes that would otherwise decrease the fitness and persistence of bacterial cells and/or populations when they are fully expressed in stationary phase. Identification of these genes and understanding the mechanisms by which their full expression results in a fitness cost might provide insights into the survival mecanisms of non-actively growing bacterial populations and might have implication for antibacterial strategies. We expect that the data presented here will inspire future studies to address these questions.

## Supporting Information

Figure S1 Central metabolic pathways controlled by σ^S^ in LB stationary phase cultures of *Salmonella*. Central metabolic pathways, including glycolysis and gluconeogenesis, the pentose phosphate pathway, the tricarboxylic acid (TCA) cycle, acetate and pyruvate metabolism are shown schematically. To assess the contribution of σ^S^ in the expression of the metabolic pathways indicated, genes differentially expressed with a p value of less than 0.05 in the wild-type and Δ*rpoS* strains of *Salmonella* were considered (Dataset S2). Genes showing differential expression with p<0.001 are indicated in bold face. Genes in red and green were negatively and positively controlled by σ^S^ respectively. Genes in black did not show differential expression in the wild-type and Δ*rpoS* strains.
(TIF)

Figure S2 Metabolic pathways controlled by σ^S^ in LB stationary phase cultures of *Salmonella*. Schematic representation of pathways controlled by σ^S^. (A) degradation of N-acetylneuraminate, N-acetyl-β-D-mannosamine and N-acetyl-D-glucosamine, (B) 4-hydroxyphenylacetate catabolism, (C) L-arabinose degradation, (D) propionate degradation, (E) Glycogen biosynthesis and degradation, (F) galactose degradation, (G) Ethanolamine utilization, (H) trehalose biosynthesis and degradation. (I) Glycine metabolism, (J) Glutamine transport and metabolism, (K) Glutathione metabolism, (L) Aspartate degradation, (M) L-serine degradation, (N) L-cysteine degradation and hydrogen sulfite biosynthesis. See also legend of Figure S1.
(TIF)

Table S1 Bacterial strains used in this study.
(DOC)

Table S2 Oligonucleotides used in this study.
(DOC)

Table S3 Coordinates in the ATCC14028 genome of the sRNAs annotated in SL1344.
(XLS)

Dataset S1 Differential gene expression in wild-type and Δ*rpoS* strains.
(XLS)

Dataset S2 Annotation of σ^S^-controlled genes (from Dataset S1, p<0.05). Genes differentially expressed in the wild-type strain and the Δ*rpoS* mutant with p<0.001 are indicated in bold face.
(XLS)
