# Supplementary figures and images for: Expanding the RpoS/σS-Network by RNA Sequencing and Identification of σS-Controlled Small RNAs in Salmonella
Source: PLoS One. 2014 May 8;9(5):e96918. doi: 10.1371/journal.pone.0096918 (PMC4014581; doi:10.1371/journal.pone.0096918)

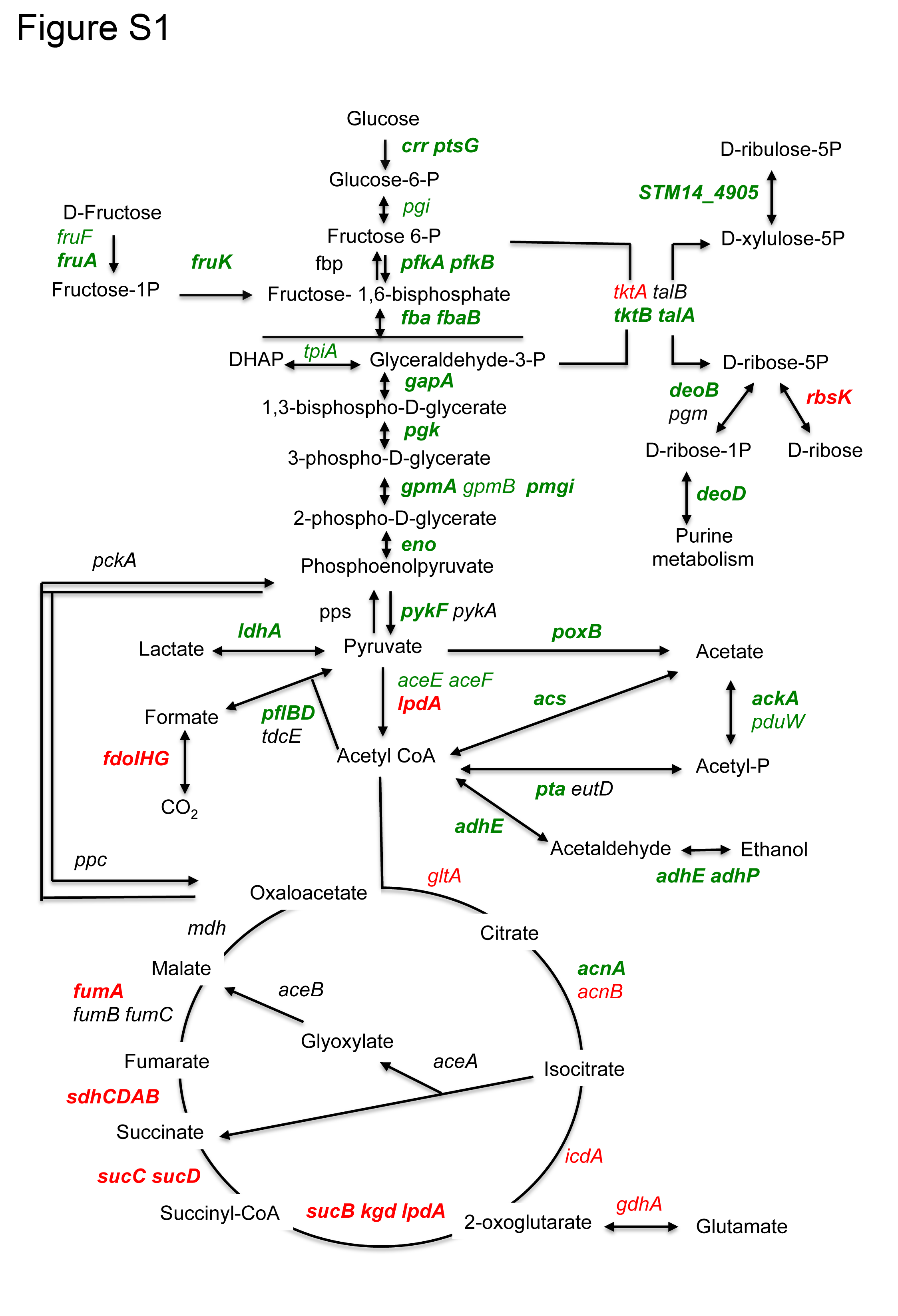

Supplement: Figure S1 — Central metabolic pathways controlled by σS in LB stationary phase cultures of Salmonella. Central metabolic pathways, including glycolysis and gluconeogenesis, the pentose phosphate pathway, the tricarboxylic acid (TCA) cycle, acetate and pyruvate metabolism are shown schematically. To assess the contribution of σS in the expression of the metabolic pathways indicated, genes differentially expressed with a p value of less than 0.05 in the wild-type and ΔrpoS strains of Salmonella were considered (Dataset S2). Genes showing differential expression with p<0.001 are indicated in bold face. Genes in red and green were negatively and positively controlled by σS respectively. Genes in black did not show differential expression in the wild-type and ΔrpoS strains. (TIF) [file pone.0096918.s001.tif]

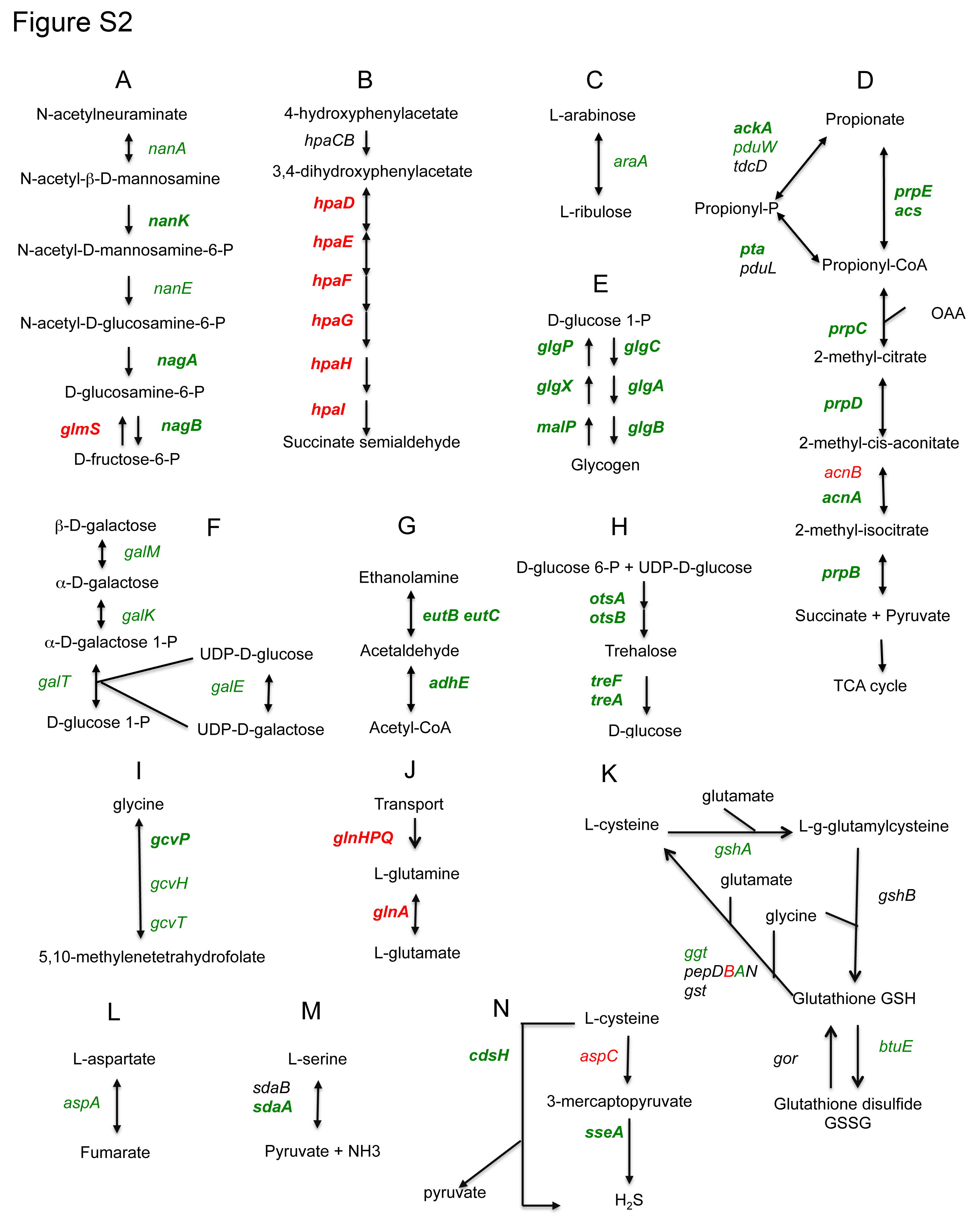

Supplement: Figure S2 — Metabolic pathways controlled by σS in LB stationary phase cultures of Salmonella. Schematic representation of pathways controlled by σS. (A) degradation of N-acetylneuraminate, N-acetyl-β-D-mannosamine and N-acetyl-D-glucosamine, (B) 4-hydroxyphenylacetate catabolism, (C) L-arabinose degradation, (D) propionate degradation, (E) Glycogen biosynthesis and degradation, (F) galactose degradation, (G) Ethanolamine utilization, (H) trehalose biosynthesis and degradation. (I) Glycine metabolism, (J) Glutamine transport and metabolism, (K) Glutathione metabolism, (L) Aspartate degradation, (M) L-serine degradation, (N) L-cysteine degradation and hydrogen sulfite biosynthesis. See also legend of Figure S1. (TIF) [file pone.0096918.s002.tif]
