# Supplementary material for: Expanding the RpoS/σS-Network by RNA Sequencing and Identification of σS-Controlled Small RNAs in Salmonella
Source: PLoS One. 2014 May 8;9(5):e96918. doi: 10.1371/journal.pone.0096918 (PMC4014581; doi:10.1371/journal.pone.0096918)
Supplement: Table S1 — Bacterial strains used in this study. (DOC) [file pone.0096918.s003.doc]

Table S1

| **Strain** | **Characteristics** | **Source or reference** |
| --- | --- | --- |
| ATCC14028 | *Salmonella enterica* serovar Typhimurium, wild-type strain | American Type Culture Collection |
| VFC326 | ATCC14028 Δ*rpoS*::*tetRA* | This study |
| VFC331 | ATCC14028 Δ*rpoS* (scarless in frame deletion of *rpoS*) | This study |
| VFD197 | ATCC14028 Δ*sdsR*::*tetRA* | This study |
| VFD164 | ATCC14028 Δ*sraL*::*tetRA* | This study |
| VFD510 | ATCC14028 Δ*csrC*::*tetRA* | This study |
| VF7969 | ATCC14028 ΔSTM2922::Km | [1] |
| VF7975 | ATCC14028 ΔSTM2922::Km Δ*rpoS*::Cm | [1] |
| VF9356 | ATCC14028 ΔSTM2922::Km Δ*rpoS* | [1] |
| VF8082 | VF7969 *katE-lacZ* | [1] |
| VF8088 | VF7969 *katN-lacZ* | [1] |
| VFD793  VFD794 | VF7969 *astA-lacZ*  VF9356 *astA-lacZ* | [2] and this study  [2] and this study |
|  |  |  |

[1] Robbe-Saule V, Dias Lopes M, Kolb A, Norel F (2007) Physiological effects of Crl in *Salmonella* are modulated by σ^S^ level and promoter specificity. J Bacteriol 189:2976–2987.

[2] Ibanez-Ruiz M, Robbe-Saule V, Hermant D, Labrude S, Norel F (2000) Identification of RpoS (sigmaS)-regulated genes in *Salmonella enterica* serovar Typhimurium. J Bacteriol 182:5749-5756.
