# Supplementary material for: Expanding the RpoS/σS-Network by RNA Sequencing and Identification of σS-Controlled Small RNAs in Salmonella
Source: PLoS One. 2014 May 8;9(5):e96918. doi: 10.1371/journal.pone.0096918 (PMC4014581; doi:10.1371/journal.pone.0096918)
Supplement: Table S2 — Oligonucleotides used in this study. (DOC) [file pone.0096918.s004.doc]

**Table S2**

| Primer name | Sequence (5’ – 3’) | Purpose |
| --- | --- | --- |
| *sdsR*-tetFw | GCAACTGGAAACCTGGCGTCGTCATCTATTCTTAAAGGTTAAGACCCACTTTCACATT | Construction of Δ*sdsR*::*tetRA* |
| *sdsR*-tetRv | TTTCGAGGGGAAACACATAACCCATTGATTTATAATCTAAATCTAAGCACTTGTCTCCTG | Construction of Δ*sdsR*::*tetRA* |
| *sraL*-tetFw | TTTCGCTGGCGAACAGGGCGTCGTCGCTTACACTTACAGTTTAAGACCCACTTTCACATT | Construction of Δ*sraL*::*tetRA* |
| *sraL*-tetRv | AAGATGATTAACATGCACTCGGCCATCGGGCTGAGCTCACCTCTAAGCACTTGTCTCCTG | Construction of Δ*sraL*::*tetRA* |
| *csrC*-tetFw | GTATCTTGTGAGTTTACCCCAAAAGAGTAAAGTAATGCACTTAAGACCCACTTTCACATT | Construction of Δ*csrC*::*tetRA* |
| *csrC*-tetRv | ATTCAGTATAGAATTGAGGCGGAATCTAGCAGAAAGCAAGCACTAAGCACTTGTCTCCTG | Construction of Δ*csrC*::*tetRA* |
| *rpoS*-tetFw | CTAGTTCCGTCAAGGGATCACGGGTAGGAGCCACCTTATGTTAAGACCCACTTTCACATT | Construction of Δ*rpoS*::*tetRA* |
| *rpoS*-tetRv | AGGCCAGTCGACAGACTGGCCTTTTTTTGACAAGGGTACTTACTAAGCACTTGTCTCCTG | Construction of Δ*rpoS*::*tetRA* |
| *rpoS*-delFw | CTAGTTCCGTCAAGGGATCACGGGTAGGAGCCACCTTATGTAAGTACCCTTGTCAAAAAAAGGCCAGTCT | Scarless in frame deletion in *rpoS* |
| *rpoS*-delRv | CGGTAAAAAAAAGGCCAGTCGACAGACTGGCCTTTTTTTGACAAGGGTACTTACATAAGGTGGCT | Scarless in frame deletion in *rpoS* |
| CL*clpX*-Fwd | GTCCATCCGTGTATATCTGC | Quantitative real-time PCR |
| CL*clpX*-Rv | AGGTGGGTACGAATTTCATG | Quantitative real-time PCR |
| CL*csrA*-Fwd | CTCGTCGAGTTGGTGAGA | Quantitative real-time PCR |
| CL-*csrA*-Rv | TCACGATGGACAGAAACTTC | Quantitative real-time PCR |
| CL-*dksA*-Fwd | GAAGCCGCCAACTTCC | Quantitative real-time PCR |
| CL-*dksA*-Rv | GCAGGACTCGCAATAACC | Quantitative real-time PCR |
| CL-*hupA*-Fwd | CAGAACTGTCCAAAACCCA | Quantitative real-time PCR |
| CL-*hupA*-Rv | GTACCGAAACCAACCAGTT | Quantitative real-time PCR |
| CL-*hupB*-Fwd | GTTGCACTGGTAGGCTTT | Quantitative real-time PCR |
| CL-*hupB*-Rv | GCACTTTGGCAGCGG | Quantitative real-time PCR |
| CL-*ihfA*-Fwd | GAAAACGGTGAGCAGGTG | Quantitative real-time PCR |
| CL-*ihfA*-Rv | CGCCGTGCTGTAATAGG | Quantitative real-time PCR |
| CL-*ihfB*-Fwd | AACCCAGCAATCTCACATTC | Quantitative real-time PCR |
| CL-*ihfB*-Rv | ATTTCAATACGCTCGCCC | Quantitative real-time PCR |
| FNB-STnc750 | AGGCCCGACGAGGAAGGGTTTGAATTTGTTTACGGCTCAA | Antisens oligo to probe for STnc750 |
| FNB-CsrC | TCCGGACGTTTGTCTTCCTGACAATCCTGTGTCTTCGCCT | Antisens oligo to probe for CsrC |
| FNB-SraL | TTTCCCCCGACGTCAACACCCCTCATATCGAGCACGTGGT | Antisens oligo to probe for SraL |
| FNB-STnc2080 | GTGTATCCCCACCCAGTGTTTTCAGTATCGAGAGCA | Antisens oligo to probe for STnc2080 |
| FNB-sRNA10 | CCGTCTTTGAAGTGATTTAGTTCACATTCGGGTAAATCCTCA | Antisens oligo to probe for sRNA10 |
| FNB-SdsR | TCTTGGGAGAGAGCCGTGCGCTAAAAGTTGGCAT | Antisens oligo to probe for SdsR |
| FNB-RyhB1_spe_ | GTGCTTTCAGGTTCTCCGTAGGGGTTCCC | Antisens oligo to probe for RyhB1 |
| FNB-RyhB2_spe_ | ACCGAACAGGTGGGTTATAAACTCAACCACTCGGT | Antisens oligo to probe for RyhB2 |
| FNB-STnc1110 | GCCGAATCTCAACGAGATCAGACCCAAAAGTCCTGCAGCA | Antisens oligo to probe for STnc1110 |
| FNB-OmrA | AGGTTGGTGCAAGAGACAGGTACGAAGAGCGTACCGA | Antisens oligo to probe for OmrA |
| FNB-MicA | CATCGCTGAAAACAGGGATGATGATAACAAATGCGCGTCT | Antisens oligo to probe for MicA |
| CL-5S | GAGACCCCACACTACCATC | Antisens oligo to probe for 5S |
